# Prevalence of and factors associated with inappropriate *Clostridioides difficile* testing in a teaching hospital in Korea

**DOI:** 10.1186/s13756-022-01111-0

**Published:** 2022-05-13

**Authors:** Hee Bum Jo, Sin Young Ham, Jongtak Jung, Song Mi Moon, Nak-Hyun Kim, Kyoung-Ho Song, Jeong Su Park, Kyoung Un Park, Eu Suk Kim, Hong Bin Kim

**Affiliations:** 1 Division of Infectious Diseases, Department of Internal Medicine, Seoul National University Bundang Hospital, 82 Gumi-ro 173beon-gil, Bundanggu, Seongnam, 13620 Korea; 2 Department of Internal Medicine, Seoul National University College of Medicine, Seoul, Korea; 3 Department of Laboratory Medicine, Seoul National University Bundang Hospital, Seongnam, Korea; 4 Present Address: Division of Infectious Diseases, Department of Internal Medicine, Incheon Sejong Hospital, Incheon, Korea; 5 Present Address: Division of Infectious Diseases, Veterans Health Service Medical Center, Seoul, Korea; 6 Department of Laboratory Medicine, Seoul National University College of Medicine, Seoul, Korea; 7 Present Address: Division of Infectious Diseases, Department of Internal Medicine, Seoul Hospital, Soonchunhyang University College of Medicine, Seoul, Korea

**Keywords:** *Clostridium difficile*, Diarrhoea, Diagnosis, Surveys and Questionnaires

## Abstract

**Background:**

Given the increasing incidence of *Clostridioides difficile* infections in Korea, there has been an increase in inappropriate testing for *C. difficile*, which has rendered overdiagnosis of asymptomatic colonisers common. We aimed to investigate the appropriateness of *C. difficile* testing and the related factors.

**Methods:**

We retrospectively reviewed the medical records of patients who were admitted to a 1300-bed tertiary-care teaching hospital in Korea and were tested for *C. difficile* infection from September 2019 to November 2019. We performed logistic regression analysis to investigate factors related to inappropriate testing. Further, a survey was conducted on physicians to assess the knowledge and ordering patterns of *C. difficile* testing.

**Results:**

We included 715 tests from 520 patients in the analysis. Testing was classified as hospital-onset and community-onset and subclassified as appropriate and inappropriate following an algorithmic method. Among the 715 tests, 576 (80.6%) and 139 (19.6%) tests were classified as hospital-onset and community-onset, respectively. Among the hospital-onset tests, 297 (52%) were considered inappropriate. The risk of inappropriate testing increased when *C. difficile* tests were conducted in the emergency room (OR 24.96; 95% CI 3.12–199.98) but decreased in intensive care units (OR 0.36, 95% CI 0.19–0.67). The survey was conducted on 61 physicians. Internal medicine physicians had significantly higher scores than non-internal medicine physicians (7.1 vs. 5.7, *p* = 0.001). The most frequently ordered combination of tests was toxin + glutamate dehydrogenase (47.5%), which was consistent with the ordered tests.

**Conclusion:**

Almost half of the *C. difficile* tests were performed inappropriately. The patient being located in the emergency room and intensive care unit increased and decreased the risk of inappropriate testing, respectively. In a questionnaire survey, we showed that internal medicine physicians were more knowledgeable about *C. difficile* testing than non-internal medicine physicians. There is a need to implement the diagnostic stewardship for *C. difficile*, especially through educational interventions for emergency room and non-internal medicine physicians.

**Supplementary Information:**

The online version contains supplementary material available at 10.1186/s13756-022-01111-0.

## Background

*Clostridioides difficile* infection (CDI) is among the most common cause of healthcare-associated infections [1]. The prevalence CDI in Korea has increased from 1.43 per 100,000 in 2008 to 5.06 per 100,000 in 2011 [2]. Recent data showed that the burden of CDI cases in the United States decreased from 2011 through 2017 [3]; however, the burden of CDI cases in Korea has been continually increasing [4].

*C. difficile* shows various clinical presentations, which range from asymptomatic colonisation to a life-threatening infection [5, 6]. The prevalence of asymptomatic colonisation with *C. difficile* is 3% to 26% and 5% to 7% in acute care hospitals [7–9] and long-term care facilities [10, 11], respectively. Although asymptomatic colonisation is not an indication for treatment, the *C. difficile* nucleic acid amplification test (NAAT) and glutamate dehydrogenase (GDH), the currently used methods cannot discriminate between CDI and asymptomatic colonisation with *C. difficile* [12], which results in overdiagnosis and overtreatment [12–15]. Therefore, there is a need to consider the relevant clinical situations and symptoms for effective CDI diagnosis [16].

In 2017, the Infectious Diseases Society of America (IDSA) and the Society for Healthcare Epidemiology of America updated the clinical practice guidelines for CDI and recommended testing in the relevant clinical settings (unexplained and new-onset ≥ 3 unformed stools within 24 h) for improved reliability of laboratory tests. Additionally, they suggested appropriate diagnostic methods [16]. This study aimed to investigate the appropriateness of *C. difficile* testing in a Korean teaching hospital. Further, we aimed to evaluate risk factors associated with inappropriate testing. Finally, we aimed to conduct a questionnaire survey of physicians to assess their knowledge and actual ordering patterns in order to investigate physician-related risk factors for inappropriate *C. difficile* testing.

## Methods

### Study population and design

We retrospectively reviewed the electronic medical records of patients who were admitted to a 1300-bed tertiary-care teaching hospital in Korea between 1 September 2019 and 30 November 2019. Eligible patients included those who underwent *C. difficile* testing. *C. difficile* toxin enzyme immunoassay (EIA), *C. difficile* GDH EIA, *C. difficile* NAAT, and *C. difficile* culture were included as *C. difficile* tests. *C. difficile* toxin EIA was performed using VIDAS *C. difficile* A & B (bioMérieux, Marcy-l’Etoile, France) while *C. difficile* GDH EIA was performed using VIDAS *C. difficile* GDH (bioMérieux, Marcy-l’Etoile, France). *C. difficile* NAAT was conducted using the BD Max Cdiff assay (Becton Dickinson Diagnostic, MD, USA). *C. difficile* culture was performed using ChromID *C. difficile* agar (bioMérieux, Marcy-I’Eltoile, France). The exclusion criteria were as follows: age < 18 years, *C. difficile* tests performed in outpatient settings, or having undergone colostomy.

### Definitions

**Fig. 1:**
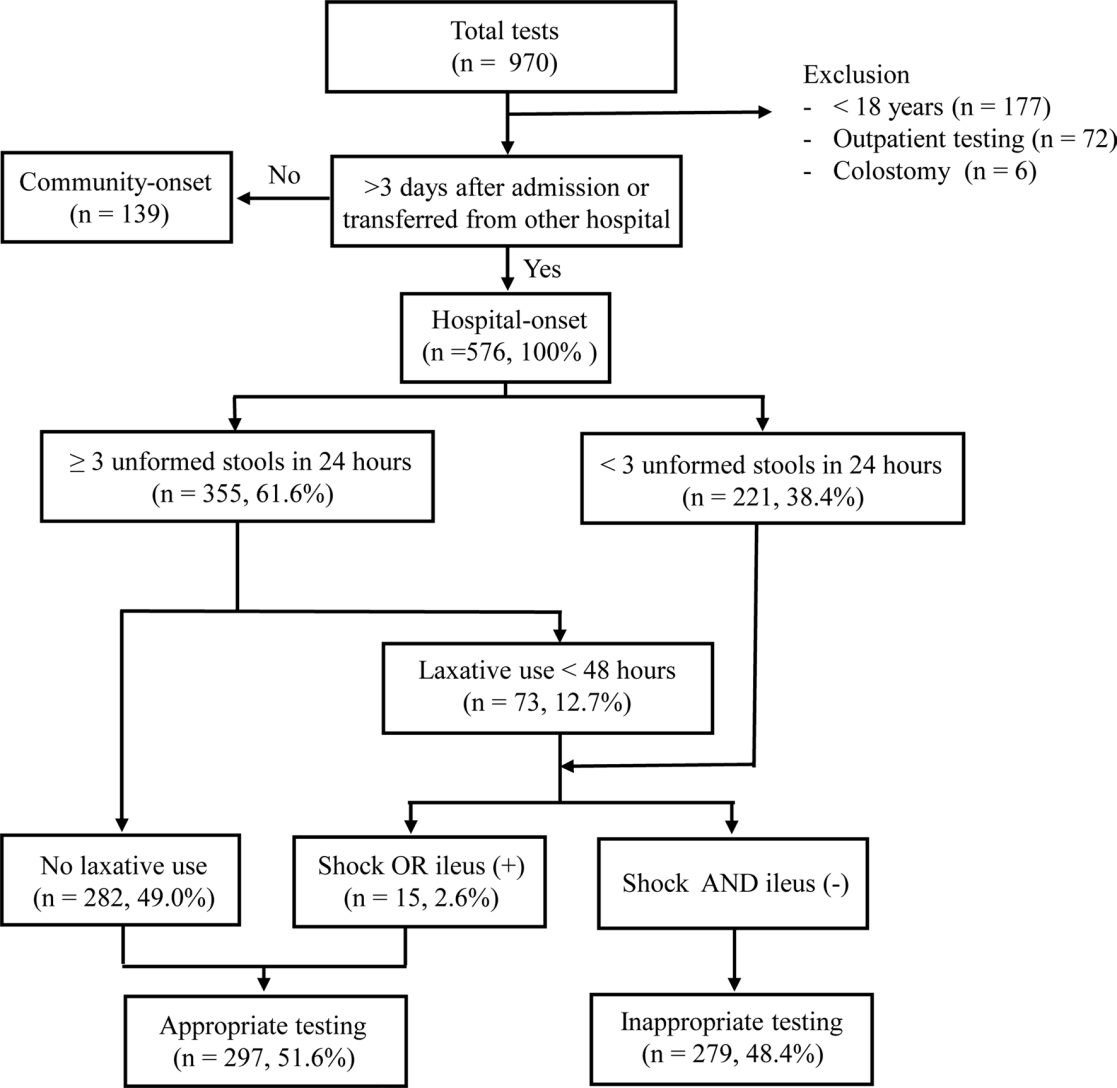
Algorithmic approach for determining the appropriateness of *C. difficile* testing

Testing for hospital-onset CDI and community-onset CDI was separately analysed. Testing for hospital-onset CDI was defined as tests performed > 3 days after admission or transfer from other hospital; testing for community-onset CDI was defined as tests performed ≤ 3 days after admission [16]. The appropriateness of CDI tests was defined using an algorithmic method (Fig. 1). The algorithm was created based on the IDSA guidelines for *C. difficile* infection [16]. Tests were defined as appropriate if the patients had new-onset, three or more unformed stools within 24 h, and did not use laxatives. Further, tests that did not meet the aforementioned criteria were considered appropriate if the patients had shock or ileus. In community-onset CDI, tests conducted on a patients who had no history of antibiotics use within 30 days of CDI test were considered inappropriate and others were classified according to same algorithmic method.

Shock was defined as mean arterial pressure < 65 mmHg or using a vasopressor on the testing day. Ileus was defined through simple abdominal radiography performed on the testing day and confirmed by a radiologist.

### Baseline characteristics

Comorbidity was determined using the Charlson comorbidity index [17]. Patients were classified into three categories based on the location of the order: general ward, intensive care unit, and emergency room. Physicians who ordered *C. difficile* testing were classified as internal medicine and non-internal medicine physicians since almost half of *the C. difficile* tests were ordered by internal medicine physicians. Further, we assumed that internal medicine physicians and non-internal medicine physicians showed different clinical practices regarding CDI-associated symptoms.

White blood cell count of 15,000 cells/mL and serum creatinine level of 1.5 mg/dL were used to indicate severe infection according to the severe CDI criteria in IDSA guidelines [16]. Fever was defined as a body temperature of ≥ 38.3 °C on the test day. The laxatives included bisacodyl, lactulose, magnesium citrate, magnesium oxide, polycarbophil calcium, polyethylene glycol, prucalopride, psyllium, senna, and sodium picosulfate. Laxative use was defined as the administration of these agents within 48 h of testing. Antibiotic use was defined as oral or intravenous administration of antibiotics within 30 days of testing. Gastrointestinal surgery included surgery of the stomach, small intestine, and large intestine within 30 days of testing.

### Questionnaire

A survey was conducted on physicians who ordered *C. difficile* testing between September 2019 and November 2019 to assess their knowledge and ordering patterns. The survey included ten questions evaluating knowledge and one question assessing the test ordering patterns (Additional file 1: Method S1). Knowledge assessment was performed on a 0–10 scale.

### Statistical analysis

Continuous and categorical variables are expressed as medians with interquartile ranges and frequencies with percentages, respectively. Between-group comparisons of continuous and categorical variables were performed using the Mann–Whitney and chi-square tests, respectively. Risk factors associated with inappropriate testing were investigated through multivariate logistic regression analysis using all significant variables on univariate analysis as well as sex and age. The questionnaire results were classified according to physician speciality (internal medicine physicians vs. non-internal medicine physicians), with comparisons using Student’s t-test. For further analysis of between-speciality differences in the scores, the scores were categorised into quartiles; further, the proportion of participants with the highest quartile was compared. The correct rate of each question was expressed as a percentage, with between-speciality comparisons being performed using the chi-square test. Statistical analyses were performed using IBM SPSS Statistics for Windows, ver. 22.0 (IBM Corp., Armonk, NY, USA). Statistical significance was set at P < 0.05.

## Results

During the study period, 970 *C. difficile* tests were performed in 691 patients. Among them, we excluded patients aged < 18 years (n = 177), patients who underwent *C. difficile* testing in an outpatient setting (n = 72), and patients who underwent colostomy (n = 6). Finally, 715 tests from 520 patients were included (Fig. 1). Among them, 576 (80.6%) and 139 (19.6%) tests were classified as hospital-onset and community-onset, respectively.

In the hospital-onset setting, 297 (51.6%) and 279 (48.4%) tests were classified as appropriate and inappropriate, respectively. Table 1 shows the baseline demographic and clinical characteristics in the hospital-onset setting. There were no significant between-group differences in age or sex. The appropriate testing group had a significantly higher median Charlson comorbidity score than the inappropriate testing group (5 [interquartile range (IQR)] 3–8) vs. 4 (IQR 3–7); *p* = 0.040). In the appropriate testing group, 243 (81.8%), 53 (17.8%), and 1 (0.3%) tests were conducted in the general ward, intensive care unit, and emergency room, respectively, while the corresponding values in the inappropriate testing group were 242 (86.7%), 23 (8.2%), and 14 (5.0%) (*p* = 0.106, 0.001, < 0.001, respectively). Compared with the inappropriate testing group, the appropriate testing group had a significantly higher proportion of patients with unformed stool ≥ 3 times within 24 h (100% vs. 20.8%; *p* < 0.001), shock (11.1% vs. 5.0%; *p* = 0.008), and ileus (11.4% vs. 5.7%; *p* = 0.015), as well as a lower proportion of patients who used laxative within 48 h (5.1% vs. 37.3%; *p* < 0.001). The appropriate testing group had a significantly higher proportion of internal medicine physicians than that in the inappropriate testing group (72.4% vs. 63.4%, *p* = 0.021). There were no between-group differences in the proportion of patients with indicators for severe infection, albumin < 3 g/dl, fever, history of gastrointestinal surgery within 30 days, history of antibiotic use over the past 30 days, transfer from other hospital. There was no difference between the two groups in the median number of days between admission and testing.

**Table 1:** Baseline characteristics of patients in whom *Clostridioides difficile* tests were performed in hospital-onset settings

Variables	Appropriate testing (n = 297)	Inappropriate testing (n = 279)	*P* value
Male	160 (53.9)	148 (53.0)	0.843
Age	73 (60.5–81.0)	72 (61.0–81.0)	0.710
Charlson comorbidity score	5 (3–8)	4 (3–7)	**0.040**
Patient location			
General ward	243 (81.8)	242 (86.7)	0.106
Intensive care unit	53 (17.8)	23 (8.2)	**0.001**
Emergency room	1 (0.3)	14 (5.0)	**< 0.001**
Physician specialty			
Internal medicine	215 (72.4)	177 (63.4)	**0.021**
Indicators for severe CDI			
White blood cell ≥ 15000 cells/mm^3^	49 (16.5)	35 (12.5)	0.179
Creatinine ≥ 1.5 mg/dl	44 (14.8)	46 (16.5)	0.581
Albumin < 3 g/dl	198 (66.7)	170 (60.9)	0.152
≥ 3 unformed stool within 24 h	297 (100.0)	58 (20.8)	**< 0.001**
Fever	77 (25.9)	64 (22.9)	0.405
Shock	33 (11.1)	14 (5.0)	**0.008**
Ileus	34 (11.4)	16 (5.7)	**0.015**
Laxative in past 48 h	15 (5.1)	104 (37.3)	**< 0.001**
Antibiotics use in past 30 days	279 (93.9)	258 (92.5)	0.484
History of gastrointestinal surgery in past 30 days	20 (6.7)	13 (4.7)	0.284
Transferred from another hospital	76 (25.6)	70 (25.1)	0.890
Days between admission and testing	11 (6–19)	10 (5–17)	0.162

Results are expressed as median (interquartile range [IQR]) for continuous variables and frequencies and percentage relative frequencies for categorical variables. *P* values were analysed using Mann–Whitney test and the chi-square test for continuous and categorical variables, respectively. Bold values indicate statistically significant differences

We performed multivariate logistic regression analysis of hospital-onset CDI with the inclusion of sex, age, and the characteristics with significant between-group differences (Charlson comorbidity score, patient location, physician speciality, shock, ileus, and use of laxatives) (Table 2). There was an increased risk of inappropriate testing in *C. difficile* tests conducted in the emergency room (odds ratio [OR], 20.06; 95% confidence interval [CI] 2.60–154.78; *p* < 0.004) and decreased risk in the intensive care unit (OR 0.33; 95% CI 0.18–0.61; *p* =  < 0.001). Tests ordered by non-internal medicine physicians were not significantly associated with inappropriate testing (OR 1.27; 95% CI 0.83–1.94; *p* = 0.271). Sex, age, and the Charlson comorbidity score were not associated with the appropriateness of *C. difficile* testing.

**Table 2:** Multivariate logistic regression analyses for inappropriate *Clostridioides difficile* testing in hospital-onset settings

Variable	Odds ratio	95% CI	*P* value
Male	1.025	0.703–1.496	0.897
Age	0.995	0.982–1.008	0.477
Charlson comorbidity score	0.970	0.904–1.040	0.390
Patient location			
General ward	1 (ref)		
Intensive care unit	0.359	0.192–0.670	**0.001**
Emergency room	24.961	3.116–199.982	**0.002**
Physician specialty			
Internal medicine	1 (ref)		
Non-internal medicine	1.299	0.847–1.992	0.230

Multivariate logistic regression analysis included all significant variables in the univariate analysis, as well as sex and age

Bold values indicate significant differences. CI, confidence interval

In the community-onset setting, 65 (46.8%) tests were appropriate and 72 tests (53.2%) were inappropriate. No risk factor was found in the multivariate logistic analysis (Additional file 1: Table S1).

**Fig. 2:**
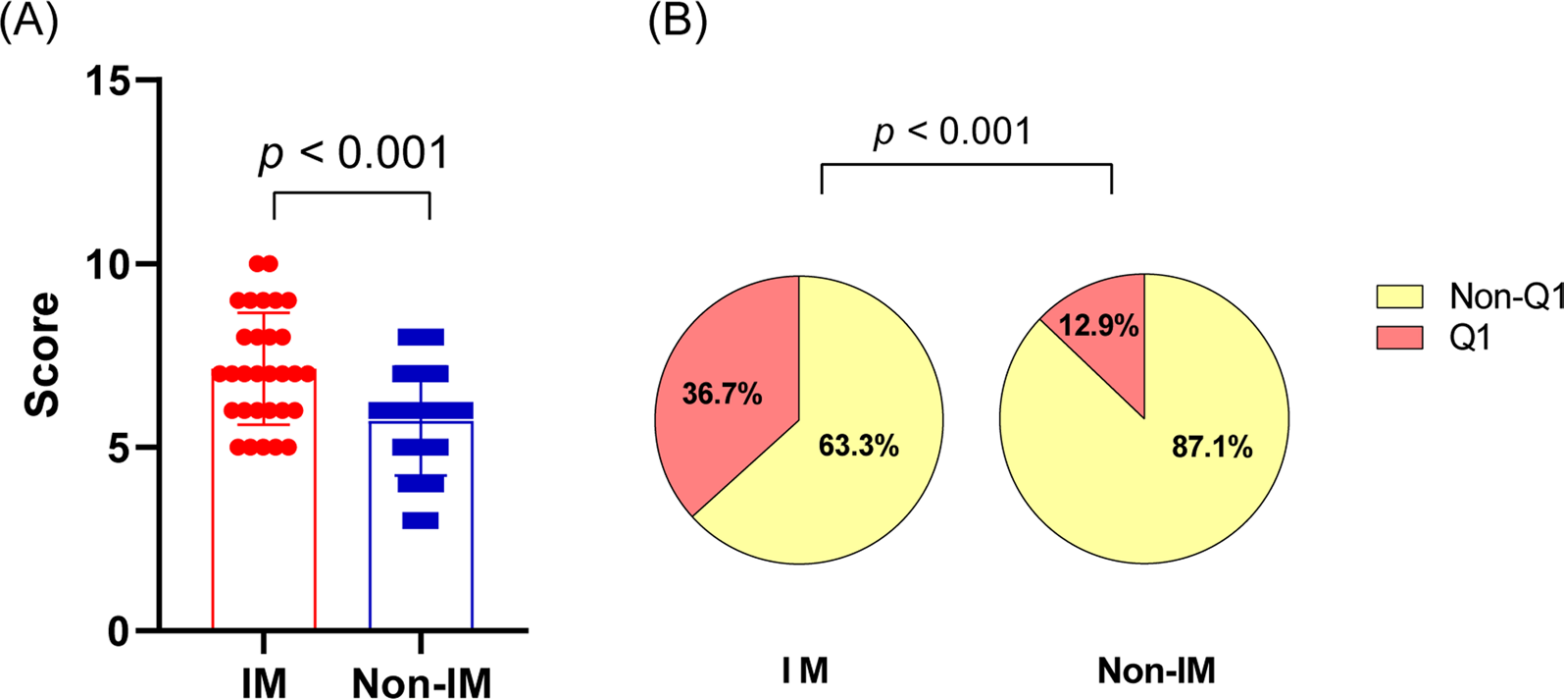
Assessment of knowledge regarding *C. difficile* testing. **A** The distribution of the knowledge score between internal and non-internal medicine physicians; **B** the proportion of participants with the highest quartile (Q1) of internal and non-internal medicine physicians. *P* values were analysed using Student’s t-test. IM, internal medicine physicians; Non-IM, non-internal medicine physicians

We conducted a survey on 61 physicians to evaluate the knowledge and performance of *C. difficile* testing. Physicians’ specialties included internal medicine (n = 30; 49.2%), general surgery (n = 18, 29.5%), emergency medicine (n = 5, 8.2%), neurosurgery (n = 4, 6.6%), neurology (n = 3, 4.9%), and others (n = 1, 1.6%). The mean score for the total population was 6.4 out of 10. Internal medicine physicians showed significantly higher scores than non-internal medicine physicians (7.1 vs 5.7, *p* = 0.001) (Fig. 2A). When the scores were classified into quartiles, internal medicine physicians had a significantly higher proportion of participants with scores in the highest quartile (score ≥ 8) than non-internal medicine physicians (36.7% vs. 12.9%, p = 0.001) (Fig. 2B). Specifically, the question regarding repeat testing (Question 9) had the lowest correct answer rate (36.7% in internal medicine physicians; 38.7% in non-internal medicine physicians; 37.7% in total population). There was a significant between-speciality difference in questions regarding culture testing (Question 4), toxin EIA with GDH EIA testing (Question 5), GDH EIA with culture testing (Question 7), and requirement of test of cure (Question 10). Additional file 1: Fig. S1 shows the percentages of correct answers for each question.

Regarding the ordering pattern of *C. difficile* testing, 29 (47.5%) physicians reported ordering Toxin EIA + GDH EIA as the initial *C. difficile* test (Table 3), while none responded with Toxin EIA + GDH EIA + NAAT, Toxin EIA + NAAT, or NAAT only. Further, 30 (49.2%) physicians reported ordering a combination of tests, including *C. difficile* culture. Similarly, the most frequent ordering pattern was toxin EIA + GDH EIA (49.7%); moreover, 274 (38.3%) tests included *C. difficile* culture.

**Table 3:** Comparison of survey responses and actual orders of *Clostridioides difficile* testing

CDI testing	Survey responses (n = 61)	Actual orders (n = 715)
Toxin EIA + GDH EIA	29 (47.5)	355 (49.7)
Toxin EIA + GDH EIA + NAAT	0 (0.0)	7 (1.0)
Toxin EIA + NAAT	0 (0.0)	8 (1.1)
NAAT	0 (0.0)	10 (1.4)
Other I (including *C. difficile* culture)	30 (49.2)	274 (38.3)
Other II (NOT including *C. difficile* culture)	2 (3.3)	61 (8.5)

Results are expressed as frequencies and percentage relative frequencies

CDI, *Clostridioides difficile* infection; EIA, enzyme immunoassay; GDH, glutamate dehydrogenase; NAAT, nucleic acid amplification test

## Discussion

Our findings demonstrated that almost half of the tests were performed inappropriately. Inappropriate testing was significantly associated with patient location. Tests performed in the emergency room increased the risk of inappropriate testing, while those performed in the intensive care unit decreased the risk. Internal medicine physicians had a significantly higher proportion of appropriate tests in univariate analysis; however, physician specialty was not statistically significant risk factor of inappropriate testing in multivariate analysis. Nevertheless, internal medicine physicians were more knowledgeable about *C. difficile* testing than non-internal medicine physicians in the questionnaire. The most frequently ordered tests were Toxin EIA and GDH EIA.

There have been inconsistent reports regarding the appropriateness of *C. difficile* testing [14, 18]. Kelly et al. [14] and Kara et al. [18] reported that 19.6% and 62% of the tests, respectively, were appropriate. Notably, Kelly et al. categorised the tests as appropriate, inappropriate, or indeterminate, where 65.5% were considered as indeterminate testing, which was mainly attributed to a lack of adequate documentation [14]. However, these findings cannot be directly compared since the studies used different criteria. Nonetheless, the findings indicate that a substantial proportion of the tests were performed in inadequate settings.

Our findings demonstrated that the location where the tests were conducted influenced the test appropriateness. Performing tests in the emergency room increased the risk of inappropriate testing. In addition to patient factors, including altered mentality, patient handoffs commonly occur in the emergency room [19], which impedes detailed history taking. Recently, several communication skills in the emergency room during physician handoffs have reduced medical errors and improved clinical outcomes [20, 21], which can be applied in CDI diagnosis. Although physician specialty was not associated with the appropriateness of testing, our survey results showed that internal medicine physicians tended to be more knowledgeable than non-internal medicine physicians. This suggests that establishing diagnostic stewardship for *C. difficile* testing, especially in emergency room and non-internal medicine physicians, could reduce unnecessary testing and treatment.

Our study showed that about 20% of CDI testing was conducted in community-onset settings. When judging by the history of antibiotics use, laxative use, shock, ileus, and, the number of diarrhoea, the proportion of appropriate CDI testing in community-onset was 46.8%. In Korea, 12.1% of CDI were reported to be community-onset CDI [22]. Similarly, in the Asia–Pacific region, 16.5% of CDI was community-associated [23]. Recent studies from the United States showed that the incidence of community-onset CDI has increased, making a considerable number of CDI cases community-onset [24, 25]. A study conducted in United States showed that 34.2% of CDI cases were community-associated CDI [26]. In this study, we could not observe the significance of the factors related to inappropriate *C. difficile* testing in community-onset setting partly because of the small number of cases. Since the importance of community-onset CDI is increasing, additional large-scale studies are needed.

We examined the ordering pattern of *C. difficile* testing by comparing the survey answers and actual orders. Approximately 50% of the physicians responded that they ordered toxin EIA + GDH EIA as the initial test for CDI diagnosis, which was comparable with the actual orders. However, NAAT was rarely used in primary testing possibly because it could be the second step of a two-step approach. IDSA and European guidelines recommend a multi-step algorithm (i.e., toxin EIA + GDH and arbitrated by NAAT) [16, 27]. In addition, the low rate of NAAT testing could be partly attributed to its recent introduction to our hospital, and thus many physicians were unaware of it. Additionally, the high costs of NAAT, about two to ten times higher than those of toxin EIA, could impede NAAT ordering [28]. Instead, many physicians ordered culture combined with conventional CDI testing. Notably, our culture method was not a toxigenic culture; therefore, it lacks diagnostic significance for CDI as it cannot distinguish between toxin-producing *C. difficile* and only colonization, although it may facilitate the analysis of epidemiological data, such as the proportion of *C. difficile* colonizers. Our findings suggest the need for diagnostic stewardship for appropriate clinical situations and proper testing methods.

Our study has several limitations. First, this was a retrospective study; therefore, the medical records may be inaccurate due to information bias. Second, this was a short-term, single-centre, non-interventional observational study; therefore, our findings may not be generalisable to other hospitals. Despite these limitations, our study has several strengths. To our knowledge, this is the first study to investigate the appropriateness and physician practice patterns of *C. difficile* testing in Korea. Further, we analysed data from a relatively large number of patients in this field. Additionally, analysis of both patient- and physician-related risk factors showed that more targeted diagnostic stewardship for emergency rooms or non-internal medicine physicians could effectively reduce unnecessary testing and treatment.

## Conclusions

Our findings showed that almost half of the *C. difficile* tests were performed inappropriately. Diagnostic stewardship for CDI should be implemented, particularly through educational interventions for those working in the emergency room and for non-internal medicine physicians. Prospective multicentre studies on the effectiveness of diagnostic stewardship are warranted.

## Supplementary Information

**Additional file 1**. **Method S1**. The questionnaire for diagnosis of *Clostridioides difficile* infection (CDI). **Table S1.** Multivariate logistic regression analyses for inappropriate *Clostridioides difficile* testing in community-onset settings. **Fig. S1.** Percentage of corrected answers to questions about knowledge of CDI testing.

## Data Availability

The datasets used and/or analysed during the current study are available from the corresponding author on reasonable request.

## Abbreviations

CDI: *Clostridioides difficile* infection
NAAT: Nucleic acid amplification test
GDH: Glutamate dehydrogenase
IDSA: Infectious Disease Society of America
EIA: Enzyme immunoassay
IQR: Interquartile range
OR: Odds ratio
CI: Confidence interval
