# Supplementary material for: Prevalence of and factors associated with inappropriate Clostridioides difficile testing in a teaching hospital in Korea
Source: Antimicrob Resist Infect Control. 2022 May 13;11:70. doi: 10.1186/s13756-022-01111-0 (PMC9107266; doi:10.1186/s13756-022-01111-0)

**Additional file 1**

Table S1. Multivariate logistic regression analyses for inappropriate *Clostridioides difficile* testing in community-onset settings

| Variable | Odd ratio | 95% CI | *P* value |
| --- | --- | --- | --- |
| Male | 1.080 | 0.341–3.418 | 0.895 |
| Age | 1.018 | 0.983–1.053 | 0.318 |
| Patient location |  |  |  |
| General ward | 1 (ref) |  |  |
| Intensive care unit | 0.065 | 0.003–1.674 | 0.099 |
| Emergency room | 0.693 | 0.041–11.448 | 0.798 |
| Physician specialty |  |  |  |
| Internal medicine | 1 (ref) |  |  |
| Non-internal medicine | 0.337 | 0.052–2.203 | 0.256 |

Multivariate logistic regression analysis included all significant variables in the univariate analysis, as well as sex and age.

Bold values indicate significant differences. CI, confidence interval

Method S1. The questionnaire for diagnosis of *Clostridioides difficile* infection (CDI)

Knowledge assessment for CDI

Question 1. Which of the following cases requires a CDI test? (multiple choices available).

□ A 70-year-old man hospitalized with a femur neck fracture and taking laxatives, diarrhoea five times per day since hospital day 4, white blood cell 14,300 /uL, C-reactive protein 8.1 mg/dL, blood urea nitrogen 10.1 mg/dL, and creatinine 1.1 mg/dL

☑ A 67-year-old woman hospitalized with acute pyelonephritis and on antibiotics, diarrhoea three times a day from hospital day 4, white blood cell 12,300 /uL, C-reactive protein 9.1 mg/dL, blood urea nitrogen 10.4 mg/dL, creatinine 0.8 mg/dL, and no laxative use

□ A 45-year-old woman, previously healthy, emergency room visit for diarrhoea five times a day, white blood cell 17,300 /uL, C-reactive protein 17.1 mg/dL, blood urea nitrogen 30.1 mg/dL, and creatinine 1.5 mg/dL

☑ An 81-year-old man, hospitalized with pneumonia and on antibiotics, diarrhoea five times a day from hospital days 5, white blood cell 16,500 /uL, C-reactive protein 23.1 mg/dL, blood urea nitrogen 30.1 mg/dL, creatinine 1.8 mg/dL, and no laxative use

☑ A 55-year-old woman with 10 days of fever, cough, and sputum on oral antibiotics, emergency room visit for new-onset abdominal pain and diarrhoea four times a day, white blood cell 12,300 /uL, C-reactive protein 5.4 mg/dL, blood urea nitrogen 10.1 mg/dL, and creatinine 0.7 mg/dL

※ The following sentence describes the results of the diagnostic test and treatment for CDI. Please check ‘yes’ if you think the description is correct, ‘no’ if you think the description is wrong, or ‘not sure’ if you are not sure.

Question 2. If *the C. difficile* toxin EIA is negative, CDI can be ruled out.

□ yes ☑ no □ not sure

Question 3. If *C. difficile* GDH is positive, CDI can be diagnosed.

□ yes ☑ no □ not sure

Question 4. If *C. difficile* culture is positive, CDI can be diagnosed.

□ yes ☑ no □ not sure

Question 5. If *C. difficile* GDH and C. difficile toxin EIA are positive, CDI can be diagnosed.

☑ yes □ no □ not sure

Question 6. If *C. difficile* NAAT and *C. difficile* toxin EIA are positive, CDI can be diagnosed.

☑ yes □ no □ not sure

Question 7. If *C. difficile* GDH and *C. difficile* cultures are positive, CDI can be diagnosed.

□ yes ☑ no □ not sure

Question 8. If pseudomembranes are not observed during colonoscopy, CDI can be ruled out.

□ yes ☑ no □ not sure

Question 9. If CDI is suspected and CDI tests are negative, repeat testing is required.

□ yes ☑ no □ not sure

Question 10. After completion of treatment for CDI, repeat tests are needed to determine whether treatment success is achieved.

□ yes ☑ no □ not sure

Ordering pattern assessment for CDI

Question 1. If you suspected CDI, please select all the primary tests you order to the patient.

□ *C. difficile* toxin enzyme immunoassay. EIA

□ *C. difficile* glutamate dehydrogenase. GDH

□ *C. difficile* culture

□ *C. difficile* nucleic acid amplification tests (NAAT)

□ No test

Fig. S1. Percentage of correct answers to questions about knowledge of CDI testing


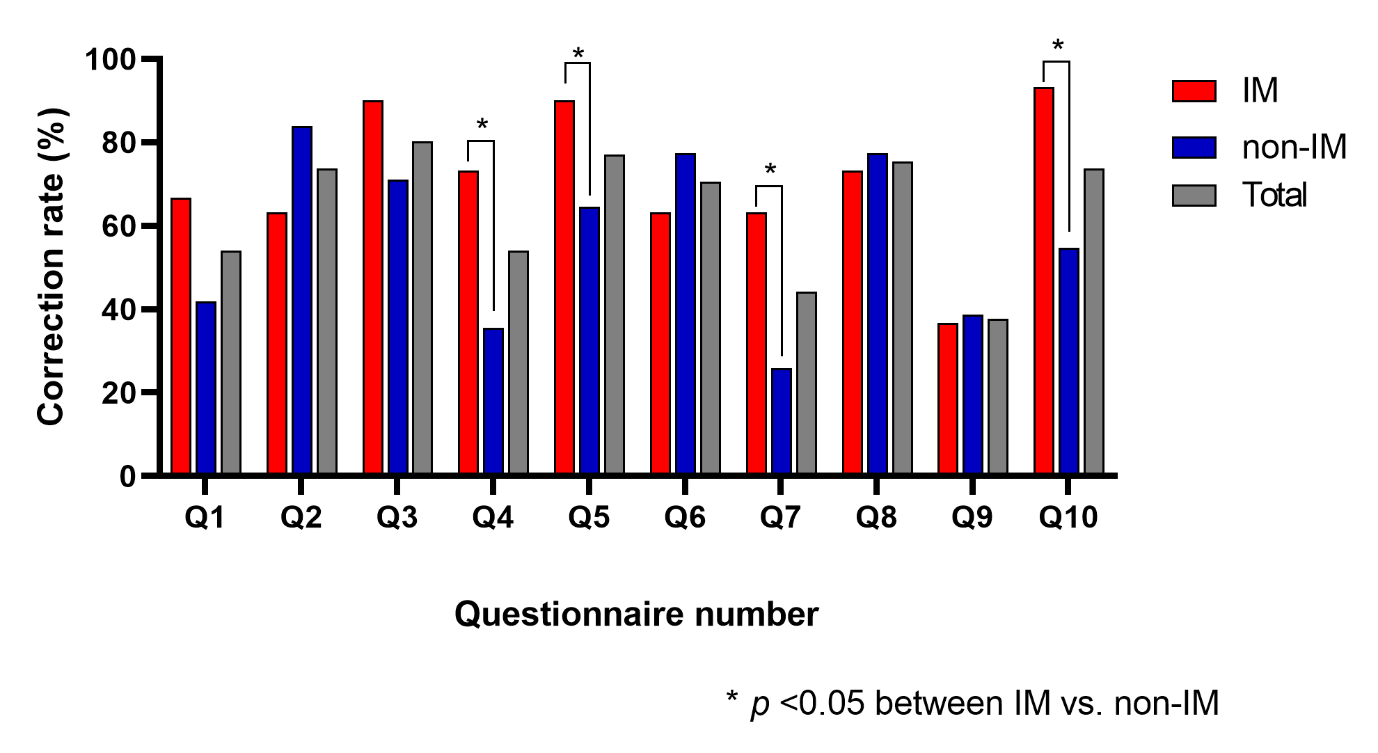

Supplement: Supplementary file 1 — Additional file 1. Method S1. The questionnaire for diagnosis of Clostridioides difficile infection (CDI). Table S1. Multivariate logistic regression analyses for inappropriate Clostridioides difficile testing in community-onset settings. Fig. S1. Percentage of corrected answers to questions about knowledge of CDI testing. [file 13756_2022_1111_MOESM1_ESM.docx]
